# Designing a Multi-Epitope Vaccine against *Chlamydia trachomatis* by Employing Integrated Core Proteomics, Immuno-Informatics and In Silico Approaches

**DOI:** 10.3390/biology10100997

**Published:** 2021-10-03

**Authors:** Sidra Aslam, Sajjad Ahmad, Fatima Noor, Usman Ali Ashfaq, Farah Shahid, Abdur Rehman, Muhammad Tahir ul Qamar, Eid A. Alatawi, Fahad M. Alshabrmi, Khaled S. Allemailem

**Affiliations:** 1 Department of Bioinformatics and Biotechnology, Government College University, Faisalabad 38000, Pakistan; sidraaslam23@gcuf.edu.pk (S.A.); fatimanoor1122@yahoo.com (F.N.); ashfaqua@gcuf.edu.pk (U.A.A.); farahshahid24@gcuf.edu.pk (F.S.); abdurrehman93@gcuf.edu.pk (A.R.); 2 Department of Health and Biological Sciences, Abasyn University, Peshawar 25000, Pakistan; sajjad.ahmad@abasyn.edu.pk; 3 College of Life Science and Technology, Guangxi University, Nanning 530004, China; 4 Department of Medical Laboratory Technology, Faculty of Applied Medical Sciences, University of Tabuk, Tabuk 71491, Saudi Arabia; eid.alatawi@ut.edu.sa; 5 Department of Medical Laboratories, College of Applied Medical Sciences, Qassim University, Buraydah 51452, Saudi Arabia; Fshbrmy@qu.edu.sa

**Keywords:** *Chlamydia trachomatis*, pan-proteomics, cholera toxin subunit B adjuvant, immune-informatics, MD simulations

## Abstract

**Simple Summary:**

*Chlamydia trachomatis* is the most common cause of blindness, ectopic pregnancy, and bacterial sexually transmitted infections. These diseases affect mostly young women but can also infect men and women of all ages. It is not difficult to treat, but it can lead to more significant health problems if left untreated. There is no licensed vaccine available for this pathogen at present. Hence, a vaccine that can control and prevent *C. trachomatis* infections is designed in this study by using different immuno-informatics approaches. However, the designed vaccine is the result of computational approaches; therefore, experimental validation is required to prove its effectiveness.

**Abstract:**

*Chlamydia trachomatis*, a Gram-negative bacterium that infects the rectum, urethra, congenital sites, and columnar epithelium of the cervix. It is a major cause of preventable blindness, ectopic pregnancy, and bacterial sexually transmitted infections worldwide. There is currently no licensed multi-epitope vaccination available for this pathogen. This study used core proteomics, immuno-informatics, and subtractive proteomics approaches to identify the best antigenic candidates for the development of a multi-epitope-based vaccine (MEBV). These approaches resulted in six vaccine candidates: Type III secretion system translocon subunit CopD2, SctW family type III secretion system gatekeeper subunit CopN, SycD/LcrH family type III secretion system chaperone Scc2, CT847 family type III secretion system effector, hypothetical protein CTDEC_0668, and CHLPN 76kDa-like protein. A variety of immuno-informatics tools were used to predict B and T cell epitopes from vaccine candidate proteins. An in silico vaccine was developed using carefully selected epitopes (11 CTL, 2 HTL & 10 LBL) and then docked with the MHC molecules (MHC I & MHC II) and human TLR4. The vaccine was coupled with Cholera toxin subunit B (CTB) adjuvant to boost the immune response. Molecular dynamics (MD) simulations, molecular docking, and MMGBSA analysis were carried out to analyze the molecular interactions and binding affinity of MEBV with TLR4 and MHC molecules. To achieve the highest level of vaccine protein expression, the MEBV was cloned and reverse-translated in *Escherichia coli*. The highest level of expression was achieved, and a CAI score of 0.97 was reported. Further experimental validation of the MEBV is required to prove its efficacy. The vaccine developed will be useful in preventing infections caused by *C. trachomatis*.

## 1. Introduction

*Chlamydia trachomatis* is an ovoid-shaped, Gram-negative, and immobile bacterium commonly known as chlamydia [1,2]. At present, around 100–150 million new victims are appearing every year globally [3,4]. Chlamydia continues to be a major pathogen among sexually transmitted pathogens and is the main cause of morbidity in the United States [5,6]. The major mode of transmission of the disease is sexual contact but can also be transmitted from an infective mother to her newborn [7]. Risk of disease is mostly seen in people aged 15–49 years old, and infections mostly occur in settings of unsafe sexual encounters [4]. Chlamydia infects the cervix, urethra, rectum, and other non-genital sites primarily through columnar epithelial cells [8,9,10,11]. Chlamydial infections in women can be serious, including cervicitis, urethritis, pelvic inflammatory disease (PID), and cervical cancer. Additionally, infections of chlamydia can induce ocular infections and can lead to blindness if left untreated [12].

The majority of *C. trachomatis* infections are asymptomatic. However, the bacteria can manifest in one of three ways: pulmonary (lungs), genitourinary (genitals), or ocular (eyes). Vaginal bleeding, genital discharge, painful urination (dysuria), and itchiness (pruritus) are some of the symptoms of genitourinary disorders [13]. When *C. trachomatis* causes trachoma in the eye, it first thickens the eyelids and then pulls the eyelashes into the eyelid [14]. When *C. trachomatis* infects the lungs as a respiratory infection, symptoms include a stuffy or runny nose, hoarseness of voice, low-grade fever, and other symptoms associated with general pneumonia [2]. *C. trachomatis* may infect pregnant women’s chorionic villi tissues latently, influencing pregnancy outcomes [15].

Treatment is determined by the location of the infection, the patient’s age, and the presence of another infection. To avoid reinfection, treatment is frequently given to both partners at the same time. Erythromycin, Azithromycin, Tetracycline, and Ofloxacin are some of the antibiotics that can be used to treat *C. trachomatis* infections [7]. Vaccination is thought to be the most effective way to lower the prevalence of *C. trachomatis* infections. It would be far less expensive and have a higher impact on global trachomatis infection control than a screening program or antibiotic treatment. Multiple types of vaccines, such as whole organisms vaccines (first generation *C. trachomatis* vaccines), subunits vaccines (second generation *C. trachomatis* vaccines), and DNA vaccines (third generation *C. trachomatis* vaccines), have been tried and were found to be ineffective [16,17,18].

The first vaccine to treat *C. trachomatis* infections was a live attenuated vaccine. The vaccine posed an immunopathology risk, and producing pure chlamydia on a large scale is difficult. The vaccine was only effective in reducing *C. trachomatis* infections in the early stages [19]. Since live vaccinations are not always safe, inactivated or killed vaccines were studied. Chemical and heating treatment was utilized for inactivation. Because of their incapacity to proliferate and induce immunity, inactivated vaccines could not give maximal protection. Sub-unit vaccines are antigen components and can overcome previous vaccine designs [4].

The complicated nature of diseases draws more attention of researchers to fully understand the pathogenesis and prognosis of the diseases and to develop effective vaccine candidates in a short time with fewer side-effects for achieving great progress in the future [20]. Tackling the major concerns that the world has been confronted with regarding global health challenges has become the need of the hour. In the last half century, knowledge regarding multi-epitope-based vaccines (MEBV) has become a thirst of the researchers who are willing and capable of designing vaccines in a short time with a small budget to meet global health challenges worldwide [21,22,23,24,25]. The emergence of rapidly endorsed and highly efficient approaches for the analysis of biological data has paved new ways to find more interesting and promising diagnostic and treatment options. The use of bio-informatics is becoming more and more common in all areas of life sciences today. Recently, there has been an explosion of new sequencing technologies that enable researchers to make important discoveries in the field of vaccine development. Usage of subtractive proteomics and immunoinformatic approaches to develop an affordable, efficient vaccine against various pathogens has recently become more attractive [26,27,28,29,30,31,32].

**Figure 1:**
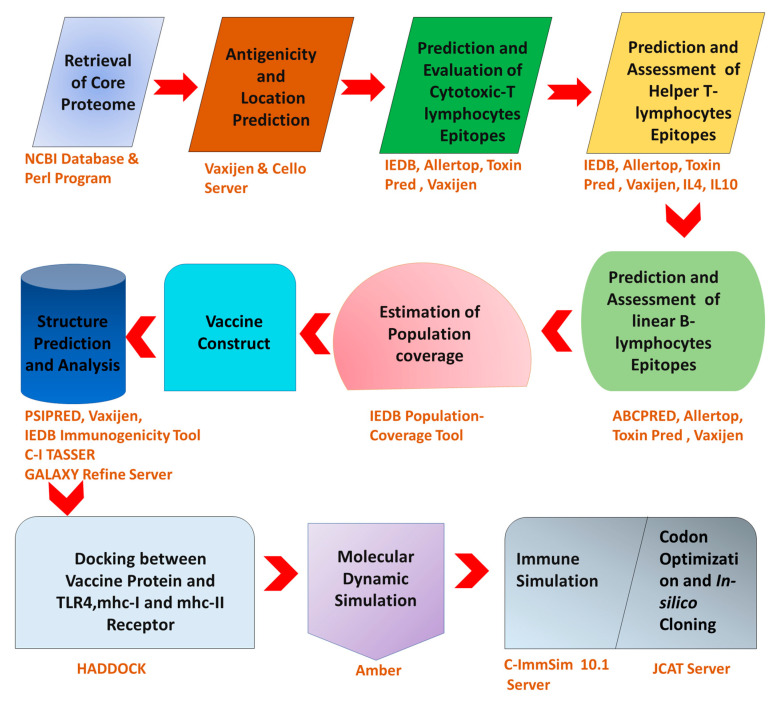
Flowchart showing the overall study workflow.

This study mainly aims to explore the core proteome of 91 *C. trachomatis* strains using reverse vaccinology, immune-informatics, and a subtractive proteomics pipeline to identify the suitable candidates for vaccine design. Further experimental research on these vaccine candidates will lead to a greater understanding of how to combat this infectious disease. Our findings will serve as a key pioneer for the researchers who seek to develop the immunogenic vaccine model against *C. trachomatis* infection. Figure 1 shows a flowchart illustrating entire method from antigen selection to vaccine design and evaluation.

## 2. Materials and Methods

### 2.1. Identification of C. trachomatis Core Proteome 

All complete 91 sequenced genomes of the *C. trachomatis* were downloaded from the NCBI genome database and investigated for the core proteome using a Perl script [33]. USEARCH was used to cluster the proteomes, and proteins with ≤50% sequence identity were discarded. The clustered sequences were then examined for the presence/absence of proteins in all input genomes and core protein sequences shared by all proteomes were considered for vaccine designing. These conserved sequences are attractive candidates for broad-spectrum vaccine design [34].

### 2.2. Subtractive Proteomics Approach

The core proteome undergoes subtractive proteome analysis for the detection of novel vaccine candidates. In subtractive proteomics, removing paralogue sequences is the first step. Cluster Database at High Identity with Tolerance (CD-HIT) is a well-known and fast program for comparing and grouping nucleotide or protein sequences to minimize the redundancy of sequences and boost the performance of sequence analysis. CD-HIT is considered as the most extensively used software to minimize the sequence redundancy. Regarding this, the core proteome was filtered using CD-HIT server at a threshold of 80%. It attempts to decrease redundancy by following a user-defined threshold of sequence identity [35]. The set of proteins obtained from the CD-HIT server was subjected to BlastP for the detection of non-homologous proteins of *C. trachomatis* against *Homo sapiens*. BlastP (Protein–protein BLAST) is an algorithm and program used to compare the query protein with the protein databases specified by users in order to retrieve the most similar protein sequences [36]. Proteins were marked as non-homologous if the protein showed query coverage of >70% and identity > 30%. To design any vaccine candidates, it is very important to have knowledge about the function of a specific protein. Sub-cellular localization prediction provides quick and worthwhile approaches to determine the function of a particular protein. Moreover, investigations have shown that localization is a key dimension for designing vaccine candidates due to the localization of proteins at multiple sites. In this regard, the CELLO server tool was used for sub-cellular localization prediction of non-homologous proteins. CELLO is a multi-class SVM based classification system, used for screening the subcellular localization of the targeted proteins [37].

Virulent Proteins are very important as they play a vital pathogenic role behind the pathogenesis of disease. All non-homologous proteins were subjected to VFDB (Virulence Factor Database) for the identification of virulent proteins [38]. The homologs marked with bit score > 100 and identity > 30% for *C. trachomatis* were regarded as virulent proteins. To predict transmembrane helices, the TMHMM server was used. TMHMM (Transmembrane Helices; Hidden Markov Model) is a program that predicts whether or not a protein contains transmembrane helices [39]. Proteins with multiple transmembrane helices were eliminated because they are difficult to express, purify, and clone, making them unsuitable for vaccine development [40]. The top antigenic proteins with 0 or 1 transmembrane helices were chosen for vaccine development.

Later, the antigenicity of virulent proteins was identified using the Vaxijen server [41]. Virulent proteins with scores > 0.5 were considered as antigenic proteins and those antigenic proteins with the best antigenicity scores were selected as a putative vaccine candidate. Aside from this, the AllerTOP server checked the allergic nature of the proteins and molecular weight was analyzed through the Protparam tool [42,43].

### 2.3. Epitopes Prediction

#### 2.3.1. HTL Epitopes

Helper T-cells have a critical role in all adaptive immune responses. HTL cells, which are the most effective cells in adaptive immunity, stimulate B-cells to secrete antibodies, assist macrophages to engulf and absorbs the pathogens, and also influence CTL cells for the killing of target parasitized cells [44]. Hence, it is critically important to predict the HTL epitope in order to generate a good immune response. The MHC-II binding prediction tool was utilized to recognize 15-mer MHC-class II T-cell epitopes [45] with a percentile rank threshold < 2. MHC-II is the main class of Major Histocompatibility Complex (MHC), present on professional antigen-presenting cells such as B cells, dendritic cell, etc. These cells play a crucial role in the initiation of immunological responses. The HTL epitope occupies a central position in the vaccine design because they produce several cytokines, such as interleukin-4 (IL-4), interferon-gamma (IFN-γ), and interleukin-10 (IL-10), causing the activation of cytotoxic T-cells and other immune cells. Hence, the IFN epitope server was utilized to predict the interferon-gamma (IFN-)-generating HTL epitopes with the Non-IFN- versus IFN model [46]. Moreover, IL-4 pred [47] and IL-10 pred servers [48] were used to predict the inducing properties of interleukin-4 (IL-4) and interleukin-10 (IL-10) using the SVM method at 0.2 and −0.3 thresholds values, respectively.

#### 2.3.2. CTL Epitopes

Most of the cytotoxic T-cells reveal T-cell receptors (TCRs), which acknowledged a particular antigen [49]. The Immune Epitope Database (IEDB) is a freely available resource that hosts various tools for epitope prediction and analysis. So, the prediction of the CTL epitopes to design a putative vaccine is important. A vaccine candidate must be immunogenic, antigenic, and free of toxins as well as allergic reactions. The IEDB MHC I tool was used to recognize 12-mer MHC-class I epitopes using the consensus method [50]. Humans were chosen as the source species for HLA alleles, and epitopes with a consensus score of <2 were chosen for future study. The human leukocyte antigen (HLA) contains multiple distinct alleles, which allow it to fine-tune the adaptive immune system. The HLA plays a critical role in the body’s immunological response against foreign substances. The IEDB-AR v.2.22 MHC-I immunogenicity tool was used for the identification of immunogenicity [51]. The antigenic characteristics of the predicted epitopes were evaluated using VaxiJen v2.0 [41]. Allergic reactions should not be produced by the vaccine components. Hence, the Allertop 2.0 server was used to forecast the allergenicity of epitopes [42]. Furthermore, Toxinpred server was employed to check their toxicity [47]. 

#### 2.3.3. LBL Epitopes

The B-Cell epitope vaccine has a vital role in adaptive immunity as it contributes to antigen-specific immunoglobin development. These epitopes have undergone further classification and have been divided into conformation and linear epitopes; however, linear epitopes are considered in the development of a vaccine. For efficient incorporation in vaccine constructs, the linear B-cell epitopes (LBL) were predicted using the ABCPred online server which works on the principle of neural networking methodology [52]. Furthermore, the toxicity, antigenicity, and allergenicity of epitopes were further tested by ToxinPred, VaxiJen v2.0, and AllerTop v1.0 servers, respectively [41,42,47].

### 2.4. World Population Coverage Analysis

Based on ethnic communities, the transmission and the expression of the HLA alleles differ, consequently helping to enhance the design of an epitope-based vaccine. In the world population, the role of the HLA allele’s distribution is necessary for multi-epitope vaccine development. IEDB-AR v2.20 examined the population coverage of the selected epitopes and their specific HLA binding alleles [53].

### 2.5. Designing and Validation of MEBV 

B-cell, HTL, and CTL were joined to form an MEBV construct with the appropriate adjuvant (immunogenic element which elevate immunogenicity in the vaccines) and linkers. Instead of large proteins or complete genomes, which are commonly employed in recombinant vaccine technology, the multi-epitope-based vaccine (MEBV) elicits immune responses based on small immunogenic sequences. As a result, this method prevents both excessive antigenic load and allergic responses in the host. Cholera enterotoxin subunit B was used as the adjuvant for the vaccine construct (accession no: P01556). An EAAAK linker was used to combine the adjuvant because it increases the stability of the overall structure. Then, CTL, LBL, and HTL epitopes were attached by an AAY linker, KK linker, and GPGPG linker, respectively.

### 2.6. Structural Analysis of MEBV Construct

The Protparam server was employed in order to evaluate the physio-chemical properties of the MEBV construct, which included the Theoretical Isoelectric Point (theoretical PI), Grand Average of Hydropathicity (GRAVY), Alphabetic Index (AI), Molecular Weight (MW), Instability index (II), etc. [54]. Furthermore, the antigenicity and immunogenicity of the MEBV construct were checked by employing the VaxiJen v2.0 server [41] and immunogenicity IEDB tool [50], respectively. AllerTop [42] was used to analyze the allergenicity of the vaccine designed with a main focus of avoiding any vaccine-related allergen reactions. SOPMA Tool evaluates the secondary structure of the MEBV construct, which is important because it is a key indicator for protein folding [55]. In addition, the solubility of the MEBV construct was checked by employing the SolPro server [56].

### 2.7. Tertiary Structure Prediction and Validation

The I-TASSER server was utilized for the modeling of the 3D structure of the MEBV construct by using various computational algorithms [57]. The I-TASSER server predicts the 3D structure of the vaccine construct based on simulations of iterative template fragment assembly and multiple-threading alignments. For determining the quality of a model, I-TASSER provides confidence scores. The 3D model obtained from I-TASSER was subjected to 3DRefine web server for the refinement of the predicted model of the vaccine. Using molecular dynamics, side chains were reconstructed by the 3DRefine web-server; then, they performed their structural repacking, and after that, final complete structural refinement was carried out [58]. For the overall quality score of the predicted structure, the ProSA-web was used; the ERRAT server was employed for the evaluation of interactions between non-bonded and atom–atom, and for the evaluation of potentially prohibited and permissible dihedral phi (ϕ) and psi (ψ) angles, respectively, a Ramachandran plot was used [59,60,61].

### 2.8. B-Cell Epitope Mapping

ABCPred and Ellipro servers of IEDB-AR v.2.22 were employed for the prediction of linear and conformational B-cell epitopes of the MEBV construct, respectively [52,62]. The Immune Epitope Database Analysis Resource (IEDB-AR) is a companion website to the IEDB that offers computational tools for predicting and analyzing B and T cell epitopes. In the ABCPred server, the amino acid sequence of the vaccine construct was taken as the input by keeping a threshold of 0.5 and a length of 14 amino acids. However, in the Ellipro tool, a 3D model of the vaccine was taken as the input and by keeping all parameters as default.

### 2.9. Molecular Docking (MEBV + TLR4, MHCI and MHCII)

When a vaccine injects into the host it interacts with host cells and triggers the immune system. To investigate this interaction, the protein–protein docking method was performed to analyze MEBV’s ability to attach with human MHCI, MCHII, and TLR4 molecules.

HADDOCK is a dynamic docking approach that uses the information to design biomolecular complexes. The HADDOCK server version 2.4 was used (https://bianca.science.uu.nl/haddock2.4/; accessed on 26 June 2021). HADDOCK (High Ambiguity Driven protein–protein DOCKing) is an integrative platform for protein–protein docking based on biophysical as well as biochemical information. MEBV was docked with along MHC I (PDB Id: 1I1Y), TLR4 (PDB Id: 4G8A), and MHC II (PDB Id: 1KG0). Their interacting residues were analyzed by PDBsum [63] and the dock complex was visualized by Pymol [64].

### 2.10. Molecular Dynamics Simulation

The molecular dynamics of docked complexes were studied using simulations. This was accomplished using the AMBER software and its numerous modules [65]. Assisted Model Building with Energy Refinement (AMBER) is the name given to a group of programs that enable users to run and analyze molecular dynamics simulations. Initially, the TLeap module was used to produce topological files and initial co-ordinates. In the TIP3P water box with 8.0 dimensions, the system was solved using the force field ff14SB [66]. The energy of the complex was minimized through the conjugate gradient for 1000 steps and the steepest descent to recover adverse conflicts. The device was then heated for 10 ps, and the algorithm Langevin Dynamics was used to maintain the temperature stability. The pressure was balanced as per the protocol. Finally, the complex was subjected to a 100 ns efficient simulation. The canonical ensemble of the simulation box inferred periodic boundary conditions. To keep the temperature stable, the algorithm Berendsen Coupling Integration was used [67]. To analyze the results, the TRAJectory (PTRAJ) module was used. Two properties were calculated and displayed using Xmgrace (Version 5.1.19. Available online: https://plasma-gate.weizmann.ac.il/Grace/ (accessed on 22 May 2021)) [68]. The root mean square deviation (RMSD) and radius of gyration (RoG) were used. Alpha carbon (C) coordinates are commonly thought to represent an amino acid’s position in 3D space. The RMSD approach analyzes the relative positions of protein carbon atoms over time by calculating the average distance between them [69]. The 3D packaging and density of the docked complexes are evaluated by RoG [70].

### 2.11. MMGBSA Binding Energy Analysis

The binding free energies of the dominant simulated complexes were calculated using MMGBSA. The MMGBSA (molecular mechanics, the generalized Born model and solvent accessibility) approach is used to elicit free energies from structural information circumventing the computational complexity of free energy simulations [71]. The initial prompt files for MEBV, TLR4, MHCI, MHCII, and complexes were analyzed and created by the ante-MMPBSA.py module. Free energy was calculated using the receptor, complex, and vaccine energies variation:

ΔGbind = (ΔGcomplex) − (ΔGreceptor + ΔGvaccine)(1)

Throughout this procedure, the energy impacts of gas-phase salvation free energy modules are shared between non-polar and polar salvation free energy modules [72]. MMGBSA calculates Gibb’s free energy, which is a term used to represent the amount of energy, symbolized by G, for each terminus, as follows:

ΔG = Egas + ΔGsolv − TS(2)

where T stands for temperature and is multiplied by S for entropy, which is calculated using normal mode analysis. When reached at the gas stage, the MM energy from the force field is frequently employed as “Egas”. This category includes van der Waals collaboration and internal and electrostatic energy.

### 2.12. Immune Simulation

C-ImmSim version 10.1 [73] is a tool for the immune simulation used to estimate immunological response of the MEBV. It performs three major simulations, namely bone marrow, thymus and lymph node. The parameters included for immune simulation are as follows: HLA (B0702, A0101, B0702, DRB1_0101, A0101, and DRB1_0101), number of steps (100), number of injections set to 1, volume (10), and random seed (12,345). The remaining parameters were deemed to be the default.

### 2.13. In Silico Cloning

Codon optimization is required for the expression of a foreign gene in a host organism based on the specific host organism. In this case, the widely used *E. coli* K12 was considered as the host. The MEBV sequence was uploaded to the JCat server (http://jcat.de/; accessed on 13 July 2021) for codon adaptation [74]. In silico cloning was performed to assure the expression of a multiepitope vaccine in an extensively employed *E. coli* pET28a (+) vector with the SnapGene v4.3 server [68].

## 3. Results

### 3.1. Core Proteome Analysis 

The core proteins are currently appreciated in the design of vaccines, as they exist in all or most of the target pathogen strains and their inclusion in the formulation of vaccines provides immune protection against wider pathogenic species. For vaccine design against *C. trachomatis*, 91 prominent pathogenic strains of *C. trachomatis* were considered (Additional File 1). The total protein count of these strains was 81,485, which was reduced to 66,696 after core proteome analysis.

### 3.2. Identification of Target Proteins

Subtractive proteomic analysis was performed to analyze the core proteome of *C. trachomatis* using different computational tools and databases. The core proteome consists of 66,696 proteins. These proteins were subjected to CD-HIT at an 80% threshold and 798 proteins were retrieved from 66,696 proteins by excluding paralog sequences. The non-redundant proteins are not important for the survival of an organism; therefore, these proteins may not be targeted directly. The identification of essential proteins that are non-homologous to host proteins are very important because these proteins are necessary for the survival of pathogens and also considered a requirement to prevent the cross binding of drugs with the host proteins [75]. BlastP was used to identify an essential protein that is non-homologous to humans. When 798 proteins were subjected to BlastP with an identity ≤30%, 547 essential proteins were distinguished that are non-homologous to humans. To obtain the information of how particular proteins perform their function, sub-cellular localization prediction was performed. Out of the total 547 targets, 210 were predicted as cytoplasmic and were excluded from the studies. The remaining 337 proteins (36 extracellular, 91 outer membranes, 136 inner membranes and 74 periplasmic) were blasted against VFDB and yielded 34 virulent proteins with a cut-off bit score >100 and sequence identity ≤30. The antigenicity of these proteins was evaluated utilizing the VaxiJen v2.0 server. Among these 34 proteins, 17 were found to be highly antigenic. In addition to this, nine proteins were found to have no transmembrane helices. Furthermore, six proteins were found to be non-allergenic and having a molecular weight of <110 kDa, making them prime candidates for vaccine development and to be taken as a target. Details of these proteins are enlisted in Table 1.

### 3.3. Epitopes Prediction

The selected target proteins were screened for CTL, HTL, and LBL epitopes. A total of 217 unique CTL epitopes with MHC-1 binding alleles were predicted. The top 11 CTL epitopes were selected, which were non-toxic, antigenic, non-allergen, and immunogenic (Table 2).

A total of 215 HTL epitopes with MHC-II binding alleles were predicted and only two HTL epitopes that were highly antigenic, IL-4 and IL-10 inducers, and IFN-γ positive were picked for MEBV designing (Table 3). 

Moreover, 315 unique LBL epitopes were forecasted and, based on their anti-toxic, immunogenicity, rich antigenicity, and non-allergenicity, the top 10 LBL epitopes were chosen for MEBV design (Table 4).

### 3.4. Population Coverage Analysis

**Figure 2:**
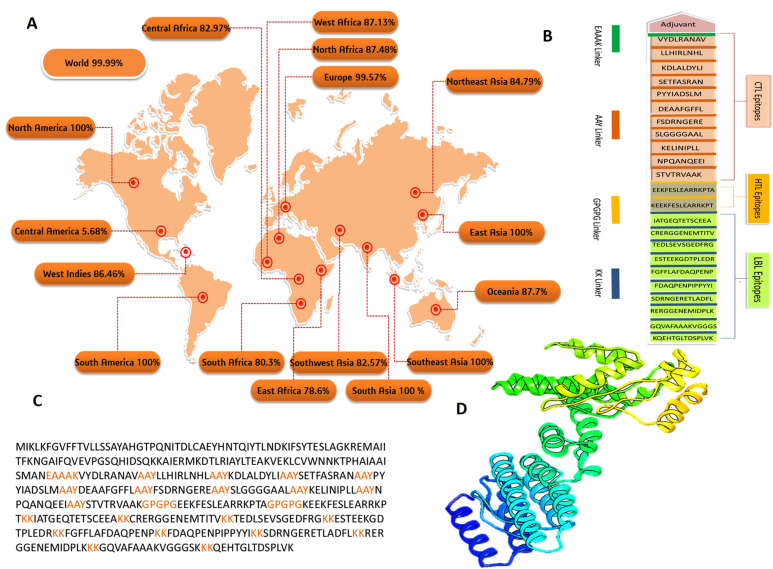
Various vaccine design analyses were conducted in this study. (**A**) Global population coverage map based on the opted T cell epitopes. (**B**) Graphical map of the designed multi-epitope-based vaccine (MEBV) construct. (**C**) The primary sequence of the MEBV, where black color represents adjuvant and epitopes and orange color represents linkers. (**D**) Tertiary structure of the MEBV.

A global population coverage study was performed on the chosen CTL and HTL epitopes, as well as their alleles. HLA alleles differ between ethnic groups and geographical regions. Consequently, these influence the construction of the MEBV. The statistical results showed a cumulative distribution of 99.9% of the world population for final epitopes (Figure 2A). Maximum population coverage was found in South Asia (100%), Southeast Asia (100%), South America (100%), East Asia (100%), and North America (100%). Central America has shown the lowest population coverage (5.68%).

### 3.5. Construction of Multi-Epitope Based Vaccine (MEBV)

Finally, a 458 amino acid long vaccine was constructed by joining multiple linkers and adjuvant in appropriate manner (Figure 2C). The 11 CTL, 2 HTL, and 10 LBL epitopes were connected to AAY, GPGPG, and KK linkers accordingly to activate an antigen-specific immune response. Furthermore, the EAAAK linker was used to attach cholera enterotoxin subunit B (236 amino acids) as an adjuvant to the first CTL epitope (Figure 2B).

### 3.6. Structural Analysis of the Vaccine Construct

Physiochemical and immunological profiling of the MEBV construct was carried out. Initially, the vaccine construct was blasted against homo-sapien proteins, and the results showed that the vaccine construct had no resemblance to any human protein. After this, a detailed study regarding the allergenicity was performed. Although antigenicity and toxicity of the MEBV designed were also performed, results have shown that the vaccine model is strongly antigenic (0.8456 at 0.5 thresholds), non-allergenic, and non-toxic. The ProtParam server was employed for the evaluation of the physiochemical properties of the vaccine construct. The molecular weight and theoretical PI were 7.59 and 50,631.33, respectively, which indicates a good antigenic nature of the vaccine construct. The instability index of MEBV is 34.57, which classifies that the vaccine construct is stable. The Aliphatic index is 71.51 which considers the relative volume held by aliphatic side chains, and the GRAVY value of the vaccine construct is −0.550, which reflects the hydrophilic nature of the MEBV construct. Lastly, the solubility of the MEBV construct was predicted using the SOLPro tool, which had a probability of 0.589196; hence, it represents that there is good solubility of the MEBV construct. Moreover, no transmembrane helices were identified in the MEBV construct. All these features indicate that the MEBV construct has a good chance of being recognized as a potential vaccine candidate. The SOPMA server was employed for secondary structure analysis of the MEBV construct. Overall, the estimation of secondary structural features showed 45.95% α-helix, 14.44% β-strands, and 31.95% random coils.

Later, I-TASSER was used to find the tertiary structure of the designed MEBV (Figure 2D). Model number 1 has been identified as the best-optimized model (c-score = −1.79). The 3DRefine server was employed for the refinement of the model. From five models refined using 3DRefine, the top first model was selected. Later, the final approval of the model was made through Ramachandran plot analysis, ProSA-web, and ERRAT. According to the Ramachandran plot analysis, 77.9% of residues were located in the favored region, 1.5% in the disallowed region, 1.8% in the generously allowed region, and 18.8% in the allowed region. Moreover, the Z score of −4.85 was predicted by the ProSA web server. The quality of non-bonded atomic interactions is determined by the ERRAT so-called ‘quality factor’, with higher scores suggesting higher quality. For high-quality models, ERRAT generates an overall quality factor of >50, and our model’s quality factor was 94.27.

### 3.7. Prediction of B Cell Epitopes of MEBV

Humoral immunity is mediated by antibodies, which are secreted by B-lymphocytes. Therefore, it has been suggested that B cell epitopes must exist within the domain of MEBV. Six conformal B cell epitopes (Additional file 2: Table S1) and 33 linear B cell epitopes (Additional file 2: Table S2) were predicted from the MEBV construct.

### 3.8. Protein–Protein Docking

A vaccine must have a high binding affinity to the host’s immune receptors, such as MHC molecules and Toll-like receptors, to elicit the proper immune responses. For stimulating the immune reaction, an adequate interaction among the molecules of the immune receptors and the antigen molecule is crucial. Thus, the HADDOCK v.2.4 server was utilized to perform protein–protein docking among the MEBV construct and human TLR4, MHCI, and MHCII. Binding scores of MEBV-MHC I, MEBV-MHC II, and MEBV-TLR4 complexes were 221.3 ± 13.2 kcal/mol, 179.4 ± 17.3 kcal/mol, and 202.6 ± 13.6 kcal/mol, respectively (Table 5). According to docking statistics, MEBV has strong binding interactions with MHCI, MHC II, and TLR4.

**Figure 3:**
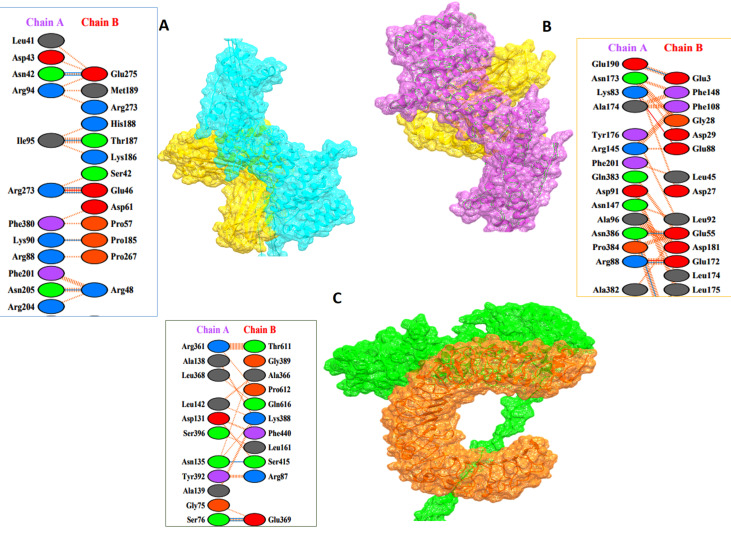
MEBV–immune receptor binding conformation and interaction analysis. Intermolecular binding mode and residue-level chemical interactions of (**A**) MEBV-MHC I; (**B**) MEBV-MHC II; (**C**) MEBV-TLR4.

A pictorial analysis of the MEBV and receptor molecule binding interactions were obtained through the PDBsum server, as well as a sketch of the interactional map among docked complexes. PDBsum provides a schematics representation of the residue interacting with binding and non-binding molecules. MEBV had eight hydrogen bonds with MHCI in the range of 3.17 Å, six hydrogen bonds with MHCII in the range of 2.58 Å, and ten hydrogen bonds with TLR4 receptor in the range of 2.82 Å. Figure 3 shows the MEBV docked conformation and atomic-level hydrogen bonding with various immunological receptors.

### 3.9. Molecular Dynamics Simulation

**Figure 4:**
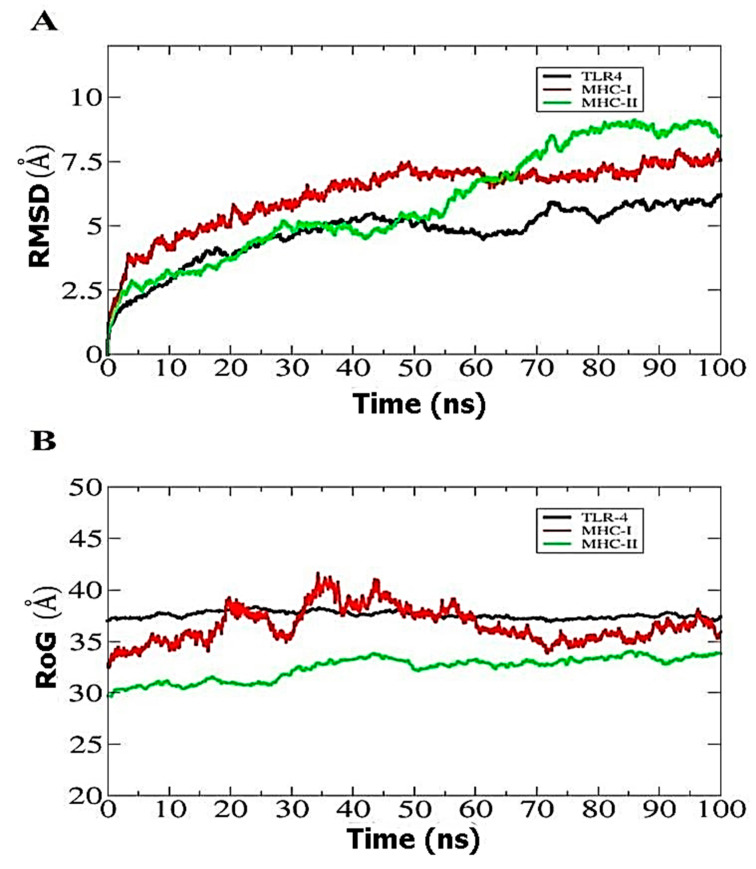
Molecular dynamics simulation-based statistical analysis to evaluate the inter-molecular stability and dynamics of the complexes. (**A**) RMSD. (**B**) RoG.

To establish the docked and structural stability of the built MEV, statistical characteristics based on the 100 ns MD simulation RMSD were computed for the docked complexes MEBV-TLR4, MEBV-MHCI, and MEBV-MHCII, as shown in Figure 4A. Both analyses used simulation trajectories. The RMSD backbone in the complexes progressively increases over time, and viewing frames at various time intervals revealed that the fluctuating plot correlates to minor structural changes generated by the MEBV due to flexible loop areas. These alterations had no effect on TLR4, MHC I, or MHC II binding, nor on the overall stability of the complexes. The MEBV-TLR4, MEBV-MHC I, and MEBV-MHC II complexes had mean RMSD values of 4.8 Å, 7.0 Å, and 8.5 Å, respectively. Complexes were next investigated by measuring the RoG in a 100 ns simulation (Figure 4B). Over a 100-ns time span, the TLR4 receptor remained stable up to 37.5 Å. The RoG MHC-II plots demonstrate that they were stable between 31.5 Å with slight deviations of 1 Å throughout a 100-ns time period. The MHC-I receptor’s RoG conformational stability with MEBV was steady at 34 Å throughout a 20-s period, then showed a small variation up to 60 ns before remaining stable up to 100 ns. These two statistical tests confirmed that the MEBV has more stable dynamics with TLR receptors than with MHC molecules, implying that the MEBV is more likely to bind to TLRs.

### 3.10. Binding Free Energies

The binding free energies of docked complexes were calculated using the MM-GBSA and MM-PBSA methods and are represented in Table 6. The Gibbs free energies of MEBV-TLR4, MEBV-MHCI, and MEBV-MHCII complexes are −121.75 kcal mol^−1^, −83.63 kcal mol^−1^, and −85.07 kcal mol^−1^, respectively in the case of MM-GBSA. In MM-PBSA, the binding free energy is −116.45 kcal mol^−1^ (MEBV-TLR4), −82.23 kcal mol^−1^ (MEBV-MHCI) and −90.81 kcal mol^−1^ (MEBV-MHCII). All the systems have an equal contribution from both van der Waals energy as well as electrostatic energy, though the former contributed more than the later. The polar energies in all three systems are non-favorable, whereas the non-polar solvation energies contributed favorably to the systems binding energy.

Based on the computed values, it appears that van der Waals energy and electrostatic energy are more useful in complex formation than the modest contribution of the non- polar fraction of the solvation energy, despite the fact that polar solvation energy is less advantageous than net energy.

### 3.11. Immune Simulation

**Figure 5:**
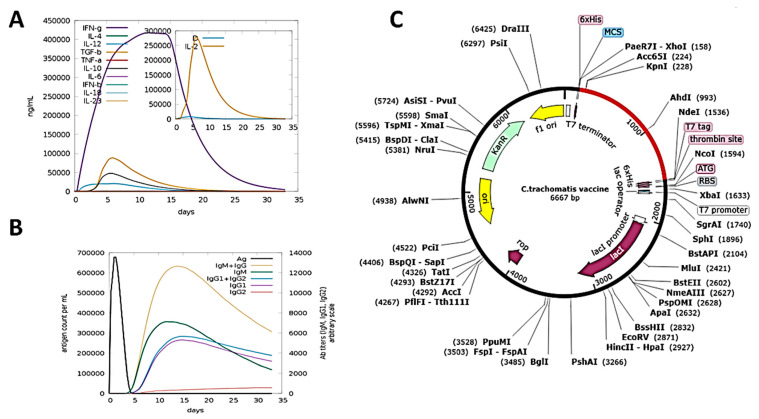
Immune simulation studies and in silico cloning of the MEBV. (**A**) Concentration of interleukin and interferon in mL/ng generated in response to MEBV. (**B**) Immunoglobulins response per mL to the presence of the MEBV antigen (**C**) Cloned MEBV sequence (colored as red) in the pET28a (+) expression vector.

The C-IMMSIM server was employed for testing the immunogenic profile of the MEBV construct. All secondary and primary immune responses contribute significantly to the immune reaction (Figure 5B). The primary reaction was described by the high concentration of IgG + IgG and IgM, followed by the secondary and primary phases of IgM, IgG1 + IgG2, and IgG1 with consequent antigen reduction. Significant cytokine and interleukin responses have also been noted (Figure 5A). All of this demonstrates the MEBV’s effective immune response and acceptance.

### 3.12. In Silico Cloning and Codon Optimization

The codon adaptation of the MEBV sequence was performed through the JCAT tool. The Java Codon adaptation tool (JCAT) serve is a novel and simple method to adapt the codon usage of the target gene to its expression host. The length of the optimum codon sequence was 1263 nucleotides. JCAT revealed that the GC content of the improved cDNA sequence was 49.30% and the CAI was 0.97%. Moreover, the adapted codon has been integrated at MCS of the *E. coli* vector pET28a (+) between the XhoI and NdeI restriction sites (Figure 5C). The clone, therefore, had a total length of 6.66 kbp.

## 4. Discussion

*Chlamydia trachomatis* is a mysterious bacterial pathogen with no vaccine available to treat ocular, pulmonary, and genital tract infections in humans [76]. Untreated genital chlamydial infection can lead to serious complications, such as infertility, pelvic inflammatory disease, and ectopic pregnancy [77,78]. The risk of HIV/AIDS and cervical dysplasia is also associated with chlamydial infection [79,80], while untreated trachoma in the eye pulls the eyelashes into the eyelid and causes blindness [14]. Although effective antimicrobial therapy is available, the number of *C. trachomatis* infections in the last decade has increased considerably due to drug resistance and a high recurrence rate. Vaccines are widely acknowledged as the most effective method of preventing *C. trachomatis* infections and related disorders, including autoimmune diseases, that are caused indirectly or directly by *C. trachomatis* infection [81,82,83]. Despite years of research and development, no vaccines that effectively prevent the aforementioned *C. trachomatis* infections are currently available. Hence, designing a vaccine that can control and prevent *C. trachomatis* infections is crucial [84,85].

Manufacturing and developing an effective live or attenuated vaccination is an expensive and time-consuming process. Aside from that, the use of classic attenuated vaccines is restricted due to certain factors, including their low ability to stimulate immune responses and a variety of adverse reactions [86]. Multi-epitope-based vaccines are preferred over traditional vaccines owing to their cost-effectiveness, improved safety, and the prospect to sensibly engineer the epitopes for amplified potency [87,88]. There are currently numerous strategies available for developing and manufacturing effective epitope-based vaccines [89,90].

In this study, subtractive proteomics was used in conjunction with reverse vaccinology and molecular docking to identify and assess antigenic peptide proteins in the core proteome of *C. trachomatis* strains. The core proteome was subjected to a subtractive proteomics pipeline to identify non-redundant, virulent, non-homologous, antigenic, and non-allergenic vaccine candidates. The number of transmembrane helices is another key criterion for excluding proteins. Because it is very difficult task to purify proteins with more than one transmembrane helix, it seems wise to exclude these proteins from the selection process [40]. The TMHMM server revealed that none of our six antigenic proteins had any transmembrane domain, implying that they are all extracellular proteins that may be fully contacted by antigen-presenting cells to trigger T- and B-cell priming and strong immune responses. To be potentially good candidates, the selected proteins must be surface-exposed and able to be recognized by the immune system. Six proteins: Type III secretion system translocon subunit CopD2, SctW family type III secretion system gatekeeper subunit CopN, SycD/LcrH family type III secretion system chaperone Scc2, CT847 family type III secretion system effector, Hypothetical protein CTDEC_0668, and CHLPN 76 kDa-like protein were identified as vaccine candidates. A slew of in vivo studies have been conducted on antigenic proteins of C. trachomatis and it is noteworthy that our five antigenic proteins, named type III secretion system translocon subunit CopD2, SctW family type III secretion system gatekeeper subunit CopN, CT847 family type III secretion system effector, and CHLPN 76 kDa-like protein, are correlated with antigenic proteins of chlamydia [91,92,93,94,95]. Following that, it gives clear evidence that all these proteins play a critical role in pathogenesis, and hence might be potential chlamydial vaccines candidates. When a persistent, substantial immune response is desired, both B and T cell epitopes must combine and generate both humoral and cellular immunity. Hence, T and B cell epitopes from vaccine candidates were forecasted and thoroughly examined. The epitopes chosen accounted for 99.9% of the global population.

To design the vaccine, HTL, CTL, and B cell epitopes were bonded to GPGPG, AAY, and KK linkers, respectively. The use of linkers in MEBV development can improve its expression, stability, and folding. The EAAAK linker is a stiff linker that has been employed in numerous studies on vaccine constructions, such as bacterial and viral diseases, particularly when separate epitopes and adjuvants are needed in the design [96]. GPGPG, AAY, and KK linkers typically consist of hydrophilic, flexible amino acids and can prevent a disruption of the domain function and folding by combining these two residues. Multi-epitope vaccines on their own cannot produce sufficient immunogenicity and require the addition of adjuvants [97]. Cholera toxin B subunit (CTB) was used as an adjuvant. CTB was studied as a traditional mucosal adjuvant with the potential to boost vaccine immunogenicity and it has been used in many previous studies [98]. Adjuvants in vaccine formulations can help protect against infection and enhance immune responses to antigens, as well as their stability, development, and duration [99]. In the functional and biochemical investigation, the solubility of the recombinant protein overexpressed inside an *E. coli* host is crucial [100]. The solubility of the MEBV protein was detected, and its ease of access to the host was confirmed. The aliphatic index and GRAVY score, respectively, are used to represent thermostability and hydrophilicity. To ensure the vaccine’s basic nature, the theoretical *pI* value was used. Furthermore, the instability index predicted in our study confirms the protein’s stability after expression, indicating that its usage capacity has improved.

The three-dimensional structure not only contains useful information about the spatial formation of major protein components, but it also aids in the investigation of protein dynamics and function, as well as interactions between ligands and other proteins [101,102]. The refinement of the MEBV construct significantly improved its desirable attributes. The Ramachandran plot shows that there are just a few residues in the disallowed area, and most are in favored regions (77.9%). The ERRAT quality factor and the Z-score for our MEBV were 94.27 and −4.85, respectively. A model with a quality factor > 50% is regarded as high-quality. The quality factor and z-score confirm our model’s high quality. The MEBV construct was used to forecast B-cell epitopes to determine whether it contained enough epitopes for antibodies to detect and latch onto [103].

The recombinant protein must be expressed in the appropriate host. *E. coli* expression systems are being developed to produce recombinant proteins. A high level of recombinant vaccine protein expression in *E. coli* K12 was the aim of codon optimization [104,105]. The high-level expression of the protein in bacteria was ensured in terms of GC content (53.63%) and codon adaptability index (0.98) values [104,106].

Since MEBV includes various epitopes (B and T cell), cellular and humoral immune responses should be triggered. IFN-β production was the highest among cytokines, and significant IL-10 and IL-2 activities were also observed. Antibodies also provided extracellular protection. A large number of active immunoglobulins, including IgM and IgG, as well as their isotypes, have been discovered that can contribute to isotype switching. Furthermore, the negligible Simpson Index (D) indicates a plausible, varied immune response because the MEBV contains a variety of B and T cell epitopes.

MEBV must have a high binding affinity for the immune receptor in order to be transported into the body successfully. The strong binding capacity of the MEBV with the MHC (MHC-I and MHC-II) molecules is necessary to elicit the immune system and to develop immunotherapy and vaccine for infectious microorganisms. These interactions initiate the naive immune response and then generate an adaptive immune response to the given epitopic antigens [107,108]. The strong interactions of MEBV with TLR4, MHC I, and MHC II were verified in MD simulation and molecular docking; the MMGBSA studies showed that this stable bonding requires very little energy. A significant number of H-bonds were observed during docking and minor fluctuations during MD simulations. These findings indicate that the MEBV can bind to immune receptors effectively.

MEBV has excellent properties that give it benefits compared with conventional vaccines, for example: (a) it contains B-cell and T-cell epitopes, and thus may be capable of generating humoral or cellular immunity inside the host; (b) it is comprised of epitopes which target various HLAs and allow the identification of several T-cell receptors, showing useful effect for a broad population; (c) a single vaccine hopefully contains several targeted proteins, as it deals with many immunogenic protein areas that tend to be combined into one peptide fragment which increases their effectiveness; (d) because the epitopes are evolved by human proteins and the rest of the unwanted proteins are removed, the risk of auto-immune illnesses may be decreased; (e) these vaccines can deliver long-lasting immunity to hosts since they are related to adjuvants; (f) these vaccines may elicit mucosal immunological responses when given orally, intranasally, or sublingually, inhibiting pathogen entry into the host’s body by inducing the generation of host-defensive B and T cells in the mucosal and systemic environments. These multi-epitope vaccines can therefore in the future become an important tool to fight pathogens. 

Shiragannavar et al. [4] and Pourhajibagher et al. [109] predicted potential epitopes for vaccine design against *C. trachomatis*, while Nunes et al. [110] identified potential vaccine antigens in their studies. Hence, no study has reported a vaccine construct yet, so the MEBV designed in this study will pave the way for future research in the field of vaccinology. Since the present study is based on an integrated computation pipeline and requires additional laboratory tests to demonstrate the safety and effectiveness of the designed vaccine.

## 5. Conclusions and Limitations 

A reverse vaccinology approach was applied on the core proteome of *C. trachomatis* strains to identify six conserved antigenic proteins. Furthermore, a multi-epitope-based vaccine (MEBV) was designed containing potential epitopes from all six antigens and evaluated using protein–protein docking and MD simulations. The designed MEBV has appropriate structural, immune, and physiochemical properties that can successfully trigger the humor and cell immune response against *C. trachomatis*. Moreover, the MEBV can easily be overexpressed in *E. coli* strain K12. The current work is, however, an outcome of an integrated vaccinomics approach. Thus, the effectiveness and tolerance of the proposed MEBV should therefore be demonstrated in laboratory tests and subsequent pharmacological trials.

## Figures and Tables

**Table 1:** Details of Antigenic Vaccine Candidates of *C. trachomatis*.

Protein Name	Accession No	Location	Antigenicity	Transmembrane Helices	Molecular Weight
Type III secretion system translocon subunit CopD2	WP_009873465	Outer Membrane	0.5273	0	55.01 kDa
SctW family type III secretion system gatekeeper subunit CopN	WP_009873547	Extracellular	0.6041	0	45.2 kDa
SycD/LcrH family type III secretion system chaperone Scc2	WP_009873911	Outer Membrane	0.6154	0	27.09 kDa
Hypothetical protein CTDEC_0668	ADI51345	Periplasmic	0.5663	0	24.45 kDa
CT847 family type III secretion system effector	WP_009873454	Extracellular	0.6101	0	19.97 kDa
CHLPN 76kDa-like protein	CPR70663.1	Extracellular	0.5456	0	68.91 kDa

**Table 2:** Final CTL epitopes used to construct MEBV.

Protein Name	Allele	Epitopes	Immunogenicity	Antigenicity
Type III secretion system translocon subunit CopD2	HLA−C *14:02, HLA−C *08:02	VYDLRANAV	0.10067	1.4953
Type III secretion system translocon subunit CopD2	HLA−B *08:01, HLA−B *14:02	LLHIRLNHL	0.19782	2.0095
SctW family type III secretion system gatekeeper subunit CopN	HLA−B *40:02, HLA−A *32:01	KDLALDYLI	0.03625	0.8238
SctW family type III secretion system gatekeeper subunit CopN	HLA−B *44:02, HLA−B *44:03	SETFASRAN	0.07931	0.892
SycD/LcrH family type III secretion system chaperone Scc2	HLA−C *14:02, HLA−A *23:01, HLA−C *07:02	PYYIADSLM	0.0456	0.6918
SycD/LcrH family type III secretion system chaperone Scc2	HLA−B *18:01, HLA−B *40:01, HLA−B *44:03, HLA−E *01:03, HLA−B *44:02, HLA−E *01:01, HLA−B *38:01, HLA−B *40:02	DEAAFGFFL	0.36517	0.6844
Hypothetical protein CTDEC_0668	HLA−C *08:02, HLA−C *05:01	FSDRNGERE	0.19962	1.889
Hypothetical protein CTDEC_0668	HLA−B *15:02, HLA−B *07:02	SLGGGGAAL	0.16588	2.1805
CT847 family type III secretion system effector	HLA−B *40:02, HLA−B *44:03, HLA−B *40:01, HLA−B *48:01, HLA−B *44:02, HLA−C *04:01	KELINIPLL	0.23346	0.625
CHLPN 76kDa-like protein	HLA−B *51:01, HLA−B *53:01	NPQANQEEI	0.02943	0.9999
CHLPN 76kDa-like protein	HLA−A *68:01, HLA−A *11:01, HLA−A *03:01, HLA−A *30:01	STVTRVAAK	0.1976	0.7123

**Table 3:** Final HTL epitopes used to construct MEBV.

Protein Name	Peptide	Allele	IL4	IL10	IFN
SctW family type III secretion system gatekeeper subunit CopN	EEKFESLEARRKPTA	HLA−DRB5 *01:01, HLA−DRB5 *01:05	Inducer	Inducer	Positive
SctW family type III secretion system gatekeeper subunit CopN	KEEKFESLEARRKPT	HLA−DRB5 *01:01, HLA−DRB5 *01:05	Inducer	Inducer	Positive

**Table 4:** Final LBL epitopes used to construct MEBV.

Protein Name	Sequence	Position	Score	Immunogenicity	Antigenicity
Type III secretion system translocon subunit CopD2	IATGEQTETSCEEA	25	0.77	0.13565	1.5065
Type III secretion system translocon subunit CopD2	CRERGGENEMTITV	1	0.67	0.33982	1.6901
SctW family type III secretion system gatekeeper subunit CopN	RERGGENEMTASGG	2	0.83	0.05641	2.1554
SctW family type III secretion system gatekeeper subunit CopN	TEDLSEVSGEDFRG	110	0.64	0.09196	1.3234
SycD/LcrH family type III secretion system chaperone Scc2	FGFFLAFDAQPENP	142	0.83	0.31428	1.0071
SycD/LcrH family type III secretion system chaperone Scc2	FDAQPENPIPPYYI	148	0.71	0.06882	1.1690
Hypothetical protein CTDEC_0668	SDRNGERETLADFL	183	0.7	0.43964	1.2628
Hypothetical protein CTDEC_0668	FSLGGGGAALDSTV	48	0.54	0.09884	1.5042
CHLPN 76kDa-like protein	GQVAFAAAKVGGGS	449	0.76	0.19157	1.0759
CHLPN 76kDa-like protein	KQEHTGLTDSPLVK	299	0.76	0.02767	1.0426

**Table 5:** Docking statistics of MEBV with immune receptors and MHC molecules.

Parameters	MEBV-TLR4	MEBV-MHCI	MEBV-MHCII
HADDOCK score	202.6 ± 13.6	221.3 ± 13.2	179.4 ±17.3
Cluster size	6	5	6
RMSD from the overall lowest-energy structure	45.4 ± 0.1	33.6 ± 0.1	9.4 ± 0.5
van der Waals energy	−33.0 ± 2.1	−48.8 ± 4.6	−60.1 ± 2.1
Electrostatic energy	−96.3 ± 21.8	−63.8 ± 10.0	−261.1 ± 24.8
Desolvation energy	−0.1 ± 2.9	−1.8 ± 1.3	−10.7 ± 3.8
Restraint violation energy	2549.5 ± 170.4	2846.2 ± 134.9	3024.6 ± 192.4
Buried Surface Area	1154.6 ± 98.4	2101.9 ± 120.2	2907.6 ± 63.1
Z-Score	−1.6	−0.8	−1.6

**Table 6:** Binding energies of the MEBV to the human receptors and MHC molecules.

Energy Parameter	TLR-4-MEBV Complex	MHC-I-MEBV Complex	MHC-II-MEBV Complex
**MM-GBSA**			
VDWAALS	−79.80	−61.96	−72.10
EEL	−71.77	−53.07	−59.00
EGB	36.45	42.58	52.13
ESURF	−6.63	−8.18	−6.10
Delta G gas	−151.57	−118.03	−131.10
Delta G solv	29.82	34.4	46.03
Delta Total	−121.75	−83.63	−85.07
**MM-PBSA**			
VDWAALS	−79.80	−61.96	−72.10
EEL	−71.77	−53.07	−59.00
EPB	43.58	41.55	49.65
ENPOLAR	−8.46	−5.75	−9.36
Delta G gas	−151.57	−118.03	−131.10
Delta G solv	35.12	35.8	40.29
Delta Total	−116.45	−82.23	−90.81

VDWAALS (van der Waals), EEL (electrostatic), EGB (polar solvation energy of MM-GBSA), ESURF (non-polar solvation energy), Delta G gas (net gas phase energy), Delta G solv (net solvation energy), Delta Total (net energy of system).

## Data Availability

The data presented in this study are available within the article.

## Supplementary Materials

The following are available online at https://www.mdpi.com/article/10.3390/biology10100997/s1, Additional File S1: Complete detail of 91 pathogenic strains of *C. trachomatis*. Additional File S2: Table S1: Conformational B cell epitopes in the MEBV predicted by ElliPro Server. Table S2: Linear B cell Epitopes in the MEBV predicted by ABCPred server.
