# Supplementary material for: Designing a Multi-Epitope Vaccine against Chlamydia trachomatis by Employing Integrated Core Proteomics, Immuno-Informatics and In Silico Approaches
Source: Biology (Basel). 2021 Oct 3;10(10):997. doi: 10.3390/biology10100997 (PMC8533590; doi:10.3390/biology10100997)
Supplement: Supplementary file 1 [file biology-10-00997-s001.zip › Additional file S2.pdf]

Table S1 Conformational B cell epitopes in the MEBV predicted by ElliPro Server

Sr. No#	Residues	Number of residues	Score
1	A:K443, A:K444, A:Q445, A:E446, A:H447, A:T448, A:G449, A:L450, A:T451, A:D452, A:S453, A:V456	12	0.981
2	A:K427, A:G428, A:Q429, A:V430, A:A431, A:F432, A:A433, A:A434, A:A435, A:K436, A:V437, A:G438, A:G439, A:G440, A:S441, A:K442	16	0.949
3	A:S396, A:D397, A:R398, A:N399, A:G400, A:E401, A:R402, A:E403, A:T404, A:L405, A:A406, A:D407, A:F408, A:L409, A:K410, A:K411, A:R412, A:E413, A:R414, A:G415, A:G416, A:E417, A:N418, A:E419, A:M420, A:I421, A:D422, A:P423, A:L424, A:K425, A:K426	31	0.772
4	A:M1, A:I2, A:K3, A:L4, A:K5, A:F6, A:G7, A:V8, A:F9, A:F10, A:T11, A:V12, A:L13, A:L14, A:S15, A:S16, A:A17, A:Y18, A:A19, A:H20, A:G21, A:T22, A:P23, A:Q24, A:N25, A:I26, A:T27, A:D28, A:L29, A:C30, A:A31, A:E32, A:Y33, A:H34, A:N35, A:T36, A:Q37, A:I38, A:Y39, A:T40, A:L41, A:N42, A:D43, A:K44, A:I45, A:F46, A:S47, A:Y48, A:T49, A:S51, A:L52, A:K55, A:R56, A:E57, A:M58, A:A59, A:I60, A:I61, A:T62, A:F63, A:K64, A:N65, A:G66, A:A67, A:I68, A:F69, A:Q70, A:V71, A:E72, A:V73, A:P74, A:G75, A:H78, A:L93, A:R94, A:Y97, A:H115, A:A118, A:A119, A:I120, A:S121, A:M122, A:A123, A:N124, A:E125, A:A126, A:A127, A:K128, A:V129, A:Y130, A:D131	91	0.699
5	A:G282, A:E284, A:F287, A:E288, A:E291, A:A292, A:R293, A:K295, A:P296, A:T297, A:K298, A:K299, A:I300, A:A301, A:T302, A:G303, A:E304, A:Q305, A:T306, A:E307, A:T308, A:S309, A:C310, A:E311, A:E312, A:A313, A:K314, A:K315, A:E318, A:I327, A:T328, A:V329, A:K330, A:K331, A:T332, A:E333, A:D334, A:L335, A:S336, A:E337, A:V338, A:S339, A:G340, A:E341, A:D342, A:F343, A:R344, A:G345, A:K346, A:K347, A:E348, A:S349, A:T350, A:E351, A:G354, A:D355, A:T356, A:P357, A:L358, A:E359, A:D360, A:R361, A:K362, A:K363, A:F364	65	0.686
6	A:G365, A:F366, A:F367, A:L368, A:A369, A:F370, A:D371, A:A372, A:Q373, A:P374, A:E375, A:N376, A:P377, A:K378	14	0.588

Table S2 Linear B cell Epitopes in the MEBV predicted by ABCPred server

Sequence	Start position	Score	Antigenicity
SMANEAAKVYDLRANA	121	0.94	1.1088
KKKQEHTGLTDSPLVK	442	0.89	1.1206
CRERGGENEMTITVKK	316	0.89	1.4302
RRKPTAGPGPGKEEKF	272	0.89	1.3671
DFRGKKESTEEKGDTP	342	0.88	2.0371
TGEQTETSCEEAKKCR	302	0.88	1.8922
YLIAAYSETFASRANA	159	0.87	0.5906
GEEKFESLEARRKPTA	262	0.86	0.7186
SCEEAKKCRERGGENE	309	0.85	2.0112
KEEKFESLEARRKPTK	283	0.85	0.7927
FFLAFDAQPENPKKFD	366	0.84	1.0855
TVTRVAAKGPGPGEEK	250	0.84	1.0499
YTESLAGKREMAIITF	48	0.83	0.6530
SEVSGEDFRGKKESTE	336	0.83	2.2665
LSSAYAHGTPQNITDL	14	0.83	0.5985
PYYIKKSDRNGERETL	390	0.82	1.4891
LGGGGAALAAYKELIN	214	0.82	0.7029
HGTPQNITDLCAEYHN	20	0.81	0.5499
QPENPIPPYYIKKSDR	383	0.79	0.6816
TVKKTEDLSEVSGEDF	328	0.79	0.7995
FLKKRERGGENEMIDP	408	0.78	0.8923
ASRANAAYPYYIADSL	169	0.75	0.6715
QVEVPGSQHIDSQKKA	70	0.71	0.7068
NGEREAAYSLGGGGAA	205	0.71	1.623
AYPYYIADSLMAAYDE	175	0.71	0.5493
KELINIPLLAAYNPQA	225	0.7	0.6806
AAYFSDRNGEREAAYS	198	0.67	0.8912
VAFAAAKVGGGSKKKQ	430	0.66	1.7192
HLAAYKDLALDYLIAA	148	0.65	0.5257
SLEARRKPTKKIATGE	289	0.54	1.0223
AYDEAAFGFFLAAYFS	187	0.53	0.5902
MIKLKFGVFFTVLLSS	1	0.53	0.9380
AQPENPKKFDAQPENP	372	0.52	0.5623
